# Development of High-Strength Aerogel Concrete

**DOI:** 10.3390/ma18051040

**Published:** 2025-02-26

**Authors:** Ourania Tsioulou, Andreas Lampropoulos, Pierfrancesco Cacciola

**Affiliations:** 1 Sustainable Engineering and Technology Education Centre (SETEC), University of Roehampton, London SW15 5PH, UK; ourania.tsioulou@roehampton.ac.uk; 2 Laboratory of Reinforced Concrete, School of Civil Engineering, National Technical University of Athens (NTUA), 15772 Zografou, Greece; 3 College of Civil Engineering, Fuzhou University, Fuzhou 350108, China; p.cacciola@brighton.ac.uk; 4 School of Architecture, Technology and Engineering, University of Brighton, Brighton BN2 4AT, UK

**Keywords:** thermal conductivity, aerogel, thermally insulated concrete

## Abstract

Aerogel is a synthetic porous ultralight material with very low thermal conductivity, and it is mainly used in buildings for external insulation in the form of blankets. In this study, the development of high-strength concrete with a partial replacement of sand with aerogel powder and aerogel beads is presented. Compressive strength, thermal conductivity, and shrinkage measurements have been conducted, and the results indicate that a replacement of sand with 30% aerogel beads leads to a high compressive strength (70 MPa) and relatively low thermal conductivity (1 W/mK) concrete.

## 1. Introduction

Worldwide, there is an ever-increasing pressure for countries, governments, and companies to become greener and reduce carbon emissions in an attempt to help prevent climate change. For this reason, in 2015, the 193 United Nations member states adopted the 2030 Agenda for Sustainable Development with 17 sustainable development goals [1].

Concrete is the most widely used construction material and has superior compressive strength, enhanced fire resistance and durability, and is a relatively ‘low cost’. Recent developments have also led to the development of novel cementitious materials with ultra-high-performance mechanical characteristics and considerably improved tensile strength properties and energy absorption. However, the main drawbacks in the use of concrete are related to the use of cement and, subsequently, the high percentage of carbon dioxide emissions as well as to the high thermal conductivity properties of concrete and the subsequent high energy consumption of the buildings. Concrete is responsible for 5% of annual anthropogenic carbon dioxide (CO_2_) production. The major contributing factor to CO_2_ emissions in the concrete industry is cement production. On average, the emissions intensity is approximately 0.8–0.9 tons of CO_2_ per ton of cement produced [2]. Most of the CO_2_ is released during the calcination process, where Calcium Carbonates (CaCO_3_) are subjected to high temperatures in a kiln, producing calcium oxides (CaO) and CO_2_. This CO_2_ is not further utilized and is released directly into the atmosphere.

One approach to reducing the environmental impact of concrete is to enhance its durability and capitalize on its positive environmental attributes. A key factor in improving energy efficiency in structures is minimizing heat loss. Studies in the literature have explored lightweight concrete, in which traditional aggregates are partially replaced by lightweight alternatives such as pumice and perlite. These studies indicate that lightweight concrete can offer superior thermal insulation properties compared to conventional concrete [2], but they also have very low strength [3,4,5,6]. In the literature, there are also some studies on the use of aerogel in concrete mixes in order to improve their thermal properties [7,8,9], but again, the compressive strength of the mixes is relatively low.

To date, research on the incorporation of aerogel beads and aerogel powder in concrete mixes is very limited [7,8,9]. Gao et al. [7] examined different concrete mix designs where sand was volumetrically substituted by aerogel powder at percentages between 10 and 60. In this research, it was found that by increasing the amount of aerogel, both thermal conductivity and compressive strength were reduced. They achieved a mix with 0.26 W/mK thermal conductivity (at 60% aerogel), which had only an 8.3 MPa compressive strength. In [8], the researchers tried to develop a high performance aerogel concrete by using some additives like concrete liquefier and silica. The results showed that in their optimum mix, the use of 60% aerogel gave 0.17 W/mK thermal conductivity, while the compressive strength was 10 MPa. In [9], Ng et al. managed to develop a mix design with 50% vol. aerogel, which gave 0.1 W/mK thermal conductivity and a 20 MPa compressive strength. These materials have been shown to produce concretes with low thermal conductivity but also reduced strength. However, no studies have yet evaluated the effectiveness of different types of aerogel in optimizing both the thermal conductivity and strength of concrete.

In this study, two types of aerogel (powder and beads) were used as partial replacements for sand alongside ground granulated blast furnace slag (GGBS) to enhance the mechanical properties of the concrete. The results demonstrate that the appropriate combination of an aerogel type and binder can yield a high-strength cementitious material with low thermal conductivity.

## 2. Materials and Methods

### 2.1. Materials

The cementitious matrix used in the current research is based on previous studies [10,11,12] on ultra-high-performance fiber-reinforced concrete and thermally insulated concrete. This mix composition has been modified by replacing part of the sand with aerogel powder and aerogel beads.

For the preparation of the mix, silica sand with a maximum particle size of 500 μm was combined with dry silica fume. The silica fume increased the density of the matrix and improved the rheological properties of the mix. Additionally, ground granulated blast furnace slag (GGBS) and cement of class 32.5 R type II were incorporated.

The cement used was Hanson Multicem, (Jewson, Coventry, UK) and was manufactured to comply with BS EN 197–1 (British Standards Institution, 2011) for a CEM II Portland limestone cement CEM II/A-LL with a strength class of 32.5 R. It is composed of a mass of 80–94% clinker and 6–20% limestone. Hanson Regen GGBS was used as the ground granulated blast furnace slag cement replacement. This was manufactured to comply with BS EN 15167–1:2006 (British Standards Institution, 2006). Elkem Microsilica Grade 940-d (Elkem, Svelgen, Norway) was used as the silica fume cement replacement. This grade of silica fume possessed a mean particle size of approximately 15 μm. The sand that was used in this experiment was T-sand by Sibelco and possessed a grain size no greater than 500 μm. Fosroc Auracast 200 (Fosroc, Dubai, United Arab Emirates) superplasticizer (SP) was used as an additive to improve the workability of the concrete mix.

The aerogel was obtained from the company Aerogel UK Ltd. (Aerogel UK Ltd., Reading, UK, 2013). This company, in turn, obtained the material from the supplier, which was imported from a South Korean producer of aerogel (Tec Co., Ltd., Seongnam-si, Republic of Korea, 2012). A low water-to-cement ratio was employed, along with a polycarboxylate superplasticizer. Using this reference mix, aerogel concrete mixes were then prepared by substituting a portion of the sand with aerogel.

Aerogel UK powder and beads are nanoporous silica aerogel with insulation performance and various applications. Aerogel powder has a thermal conductivity between 0.018 and 0.020 W/mK, and aerogel beads have a particle size of 1–6 mm and thermal conductivity between 0.016 and 0.019 W/mK. Both powder and beads are reusable, safe, and environmentally friendly.

All the examined mix designs are presented in Table 1. In this table, REF refers to the reference mix with no aerogel in it, and AC refers to the aerogel mixes. The number (10, 20, or 30) that follows the AC provides the volumetric percentage replacement of the sand with aerogel. The letter (P or B) that follows refers to the type of aerogel that was used: P for the powder aerogel and B for the beads.

The densities of all the individual materials (which were given by the material providers on the datasheet that accompanied the materials) are shown in Table 2.

### 2.2. Preparation of the Examined Specimens and Testing

#### 2.2.1. Mixing Procedure

Due to the hydrophobic nature of aerogel, a dry mixing process has been adopted. Aerogel was mixed together with cement, silica fume, GGBS, and sand for 5 min. Then, water was added slowly until the mix obtained a uniform texture and after that, superplasticizer was added as well so as to obtain a well-mixed paste.

The mix was poured into stainless steel molds (15 cubes with 50 mm side for the compressive strength measurements and 3 samples of 160 mm × 40 mm × 10 mm for the thermal conductivity measurements, per mix design), vibrated for a few seconds (around 5 s) to ensure good compaction, and kept in room temperature (23 °C and 60% humidity). After 24 h, the samples were de-molded and remained in the same room for 28 days.

#### 2.2.2. Characterization of the Mixes

For the investigation of the properties of the different concrete mixes, density measurements, compressive tests, thermal conductivity measurements, and shrinkage measurements were taken.

For the compressive tests, three 50 mm cubes of each mix and each age (1 day, 3 days, 7 days, 14 days, and 28 days) were tested in accordance with BS 1881–114:2015 standards [13].

The thermal conductivity of all the different concrete mixes was measured using a thermal conductivity analyzer (the TCi-Thermal Conductivity Analyser). The function of this analyzer is based on the modified transient source method (ASTM D7984 [14]). A one-sided interfacial heat sensor applies constant heat to the sample, and in a few seconds, the results for the thermal conductivity of the material are obtained.

For shrinkage, three prisms for each mix, with the dimensions of 160 × 40 × 40 mm^3^, were cast. Measurements at different ages (1 day, 3 days, 7 days, 14 days, and 28 days) were taken for all the samples.

## 3. Results

### 3.1. Concrete Density

**Figure 1:**
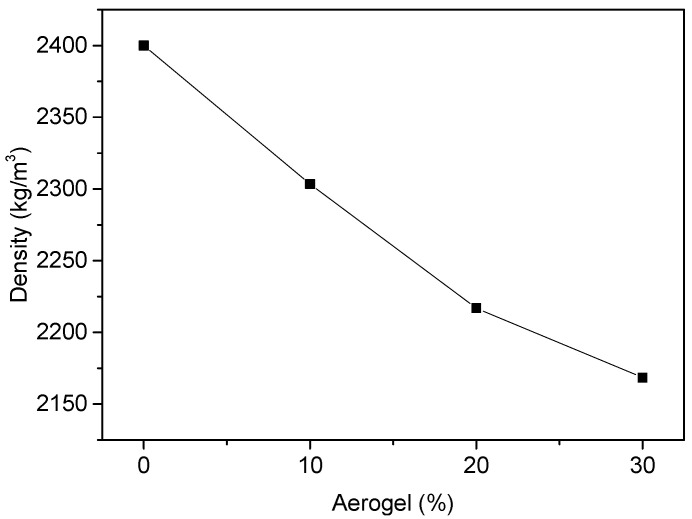
Effect of aerogel content on the density of concrete at the age of 28 days.

The results obtained for the density of the concrete samples at the age of 28 days are shown in Figure 1. In general, it can be observed that the aerogel concrete samples have less density than ordinary concrete ones. In order to calculate the density, measurements of the base, height, and width of the 50 mm^3^ samples using a digital caliper were taken. In addition to this, the mass of each sample was also measured. By dividing the mass of the concrete cube by its volume, the density can be found in kg/m^3^.

### 3.2. Compressive Strength Results

**Figure 2:**
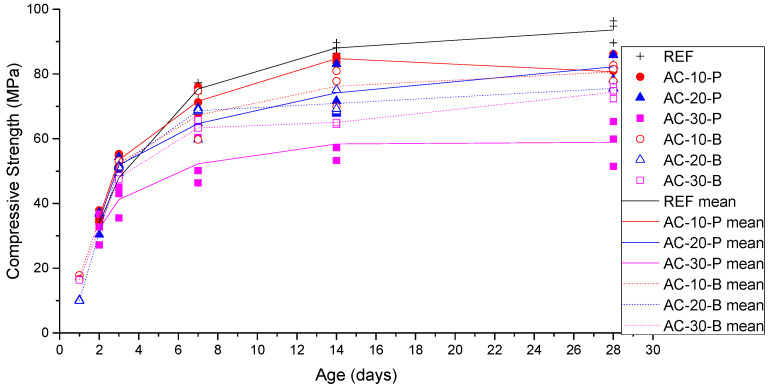
Compressive strength vs. age of the concrete mix.

The compressive strength of all the examined mixes has been examined at 2, 3, 7, 14, and 28 days after casting, and the results are presented in Figure 2, which shows the effect of age on the compressive strength of the different mixes. Based on the results of Figure 2, it is evident that the compressive strength is considerably increased during the first 14 days, as expected, and then the rate of increment is reduced as the age becomes higher.

**Figure 3:**
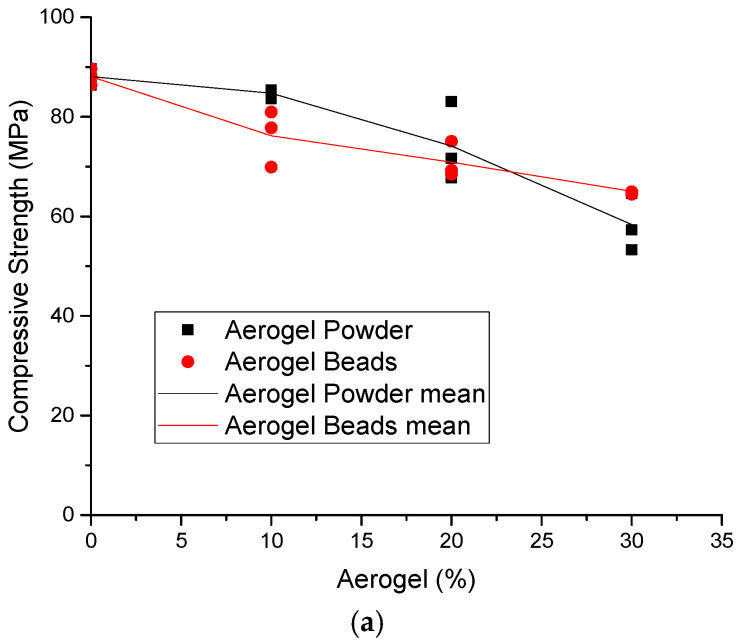

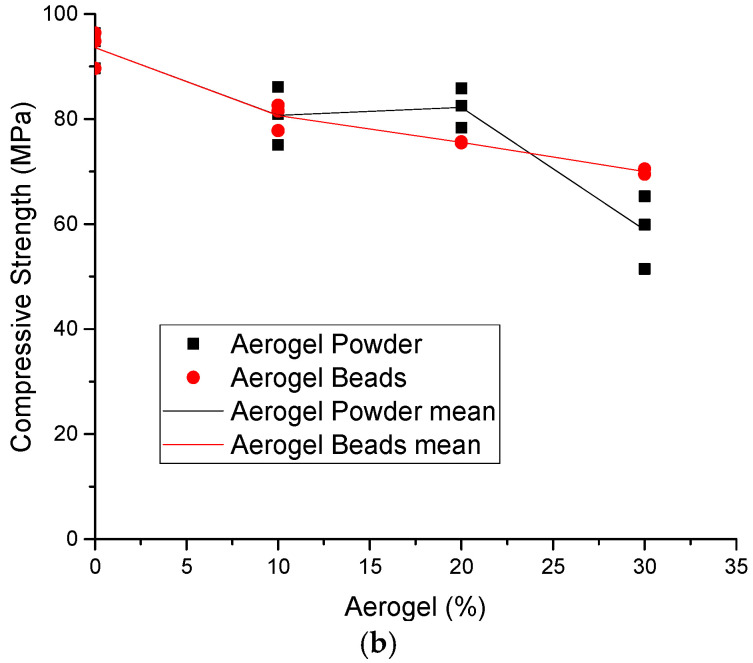
Effect of the aerogel content on the compressive strength of concrete at the ages of (**a**) 14 days and (**b**) 28 days.

**Figure 4:**
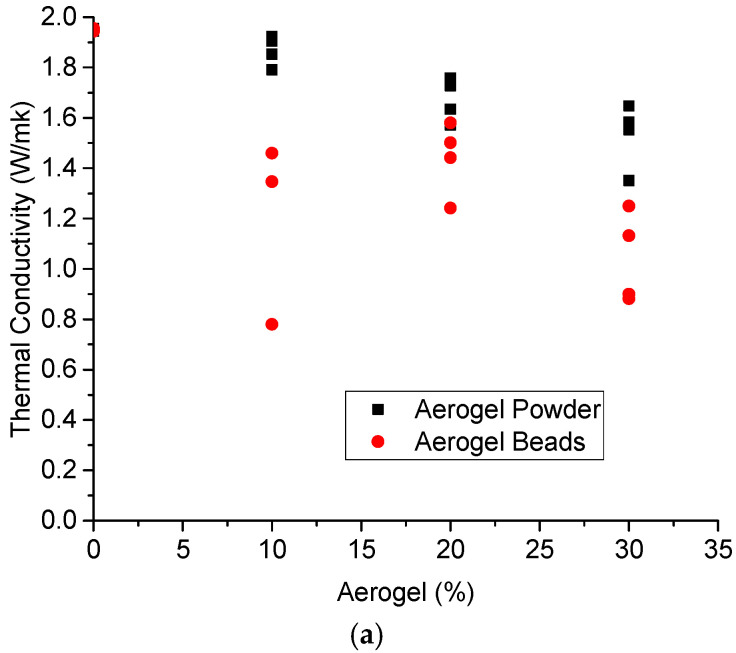

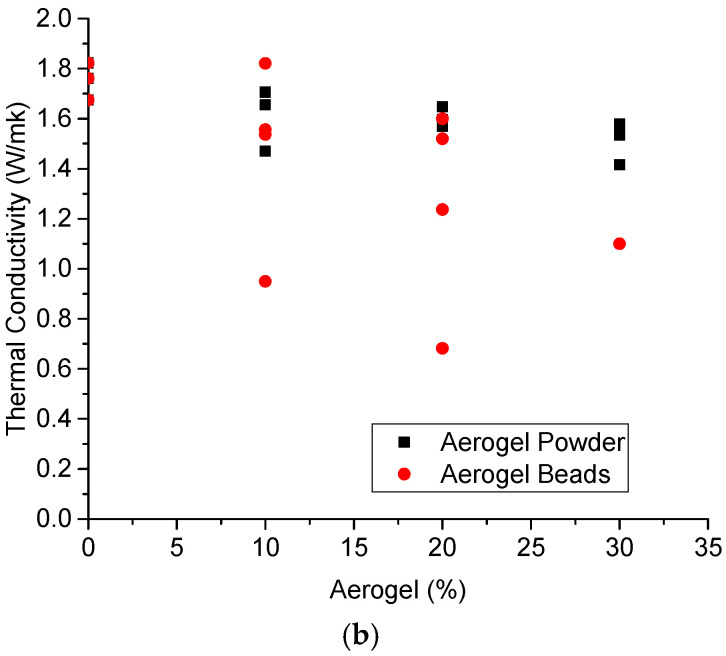
Effect of the aerogel content on thermal conductivity (W/mK) at the ages of (**a**) 14 days and (**b**) 28 days.

Figure 3a and Figure 4b present the effects of the aerogel on the compressive strength of the mixes at the ages of 14 and 28 days, respectively.

The results in Figure 3 also indicate that, in all the cases, when aerogel is added to the mix design, the compressive strength is reduced if it is compared with the reference mix. More specifically, the compressive strength is reduced as the amount of aerogel is increased. This is an expected result, as the substitution of sand with aerogel leads to lighter concrete (Figure 1). Moreover, it can be seen that apart from the case with a 30% substitution of sand with aerogel, where the aerogel bead mix provides a higher compressive strength than the respective aerogel powder one, the type of aerogel does not significantly affect the compressive strength of the mixes.

Figure 3 presents the effect of the aerogel amount on the compressive strength of the concrete mix at the age of 14 days after casting (Figure 3a) and at the age of 28 days after casting (Figure 3b). Based on these results, in both cases of aerogel powder and beads, the compressive strength is reduced as the aerogel amount is increased. This is an expected result, as according to Figure 1, the density of the mix is reduced with the increase in aerogel. Also, based on these results, it seems that the compressive strength is not considerably affected by the type of aerogel used in the current investigation (i.e., powder and beads); however, mixes with aerogel beads seem to have higher compressive strengths at the age of 28 days.

### 3.3. Thermal Conductivity

Small plates of 160 × 40 × 10 mm^3^ were cast for each mix. In the following, each of the plates was divided into four equal sections, and measurements for the thermal conductivity in each of these sections of each plate were taken.

Thermal conductivity measurements for all the examined mixes at two different ages of 14 and 28 days are presented in the following Figure 4. For each mix, four different measurements of thermal conductivity (at each of the four sections in which the plate has been divided) have been taken, and the results are presented in Figure 4.

It seems that thermal conductivity is less in the mixes where aerogel beads have been used. The spread of thermal conductivity results for the aerogel bead mixes was high, while in the aerogel powder mixes, it was very low. This observation can be explained as aerogel powder mixes are more homogeneous than the respective aerogel bead mixes. Moreover, as the proportion of aerogel in the mix increased, thermal conductivity decreased. The most significant reduction in thermal conductivity observed in this study occurred with a 30% replacement of sand by aerogel; for samples containing 30% aerogel powder, a thermal conductivity reduction of 11.7% was recorded on the 14th day, increasing to 19% on the 28th day. Similarly, specimens with 30% aerogel beads demonstrated thermal conductivity reductions of 35.8% and 31% on the 14th and 28th days, respectively.

**Figure 5:**
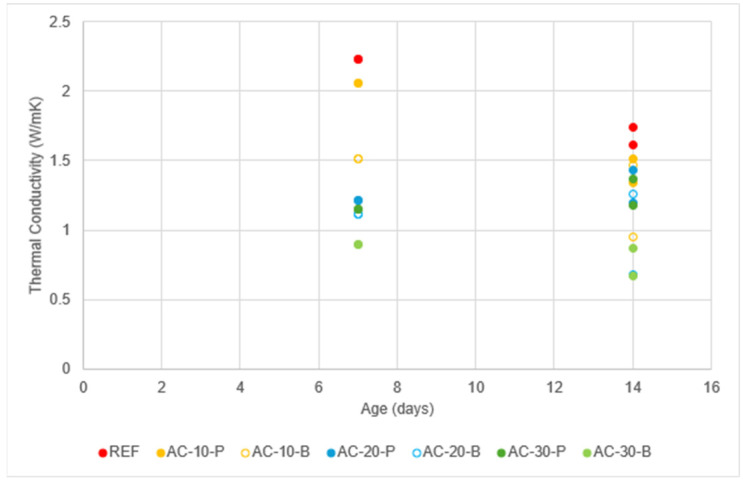
Thermal conductivity (W/mK) changes with concrete’s age.

The variation in thermal conductivity with the age of the concrete is illustrated in Figure 5. From these results (Figure 5), it can be observed that the change in thermal conductivity is not considerably affected by age. This is in agreement with previous studies on the effect of the hydration rate on the thermal conductivity properties [15].

In Table 3, which follows, a comparison between the results of the present study with the results from the literature is presented.

From the results presented in Table 3, it is obvious that the AC−30-B mix, which is presented in the current study, leads to a thermal conductivity similar to the mixes suggested in the literature but with less drop in the compressive strength if it is compared with the reference mix, and the highest compressive strength if it is compared with the other mixes suggested in the literature.

#### Distribution of Thermal Conductivity Measurements

As mentioned above, for each mix, four measurements of thermal conductivity were taken along each plate. The results at the 14-day and 28-day ages are presented in Table 4 and Table 5, respectively.

In both tables, the last line provides the coefficient of variance, CV. For the reference and the AC-powder mixes, the coefficient of variance was calculated as less than 0.1 (0.037 < CV < 0.1), while for the aerogel beads mixes, the respective values were higher than 0.1 (0.1 < CV < 0.33042).

From these results, it is obvious that the distribution of the values is much higher in the cases where aerogel beads were used. In all the cases, there is a spread in the values, as the concrete is not a homogeneous material, and also, any air void on the surface can affect the measurements, giving lower values for thermal conductivity. In the cases where beads were used, the distribution of the values was higher, as the use of beads leads to an even less homogeneous material than in the case of aerogel powder (also shown in the microstructure analyses in Figure 8). If the bead is close to the surface, the thermal conductivity measurements of that area will give very low values.

We can conclude that aerogel powder mixes are not homogeneous, and this affects the spread of the thermal conductivity measurements. In aerogel powder mixes, this spread is much lower.

### 3.4. Shrinkage

**Figure 6:**
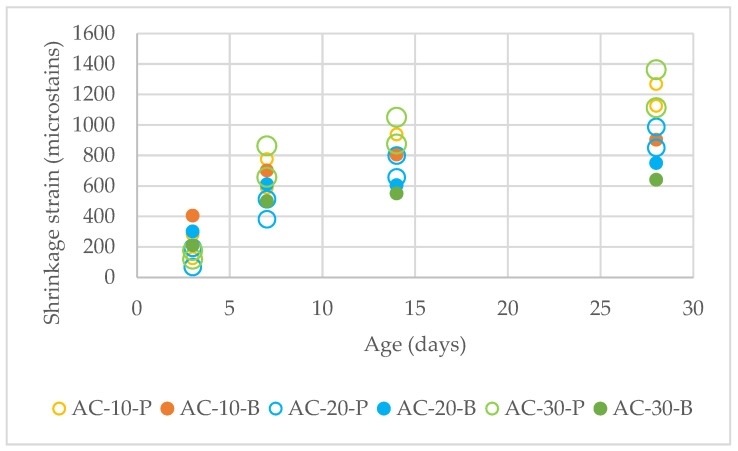
Shrinkage of different mixes.

Shrinkages of 10%, 20%, and 30% aerogel concrete mixes were measured, and the results at different ages of the mixes are presented in Figure 6.

**Figure 7:**
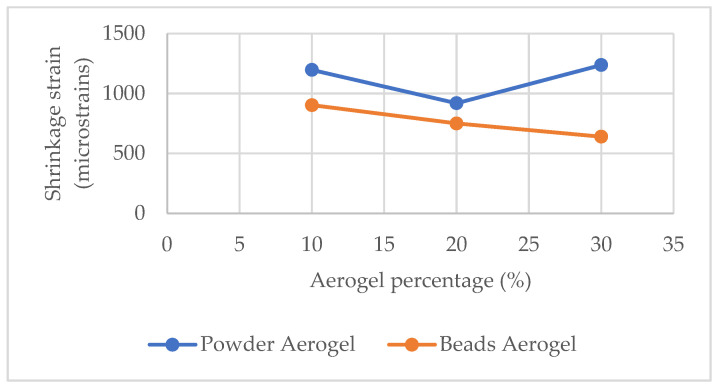
Shrinkage vs. aerogel %.

It can be seen that apart from the AC−30-P mix, in all the other cases, an increase in the aerogel amount in the mix leads to lower shrinkage. Also, aerogel powder mixes seem to have lower shrinkage than the aerogel beads mixes at an early age, while at a greater age, aerogel powder mixes have higher shrinkage than the respective aerogel beads ones, which can also be validated by the microstructure analyses presented in Figure 7 below.

Figure 7 shows a drop, apart from the case with 30% aerogel powder, in the shrinkage with the increase in the aerogel %. This can be explained due to the hydrophobic behavior of aerogel, which prevents excessive moisture loss from the concrete matrix. This reduces drying shrinkage, which is primarily caused by rapid water evaporation.

### 3.5. SEM Analyses

**Figure 8:**
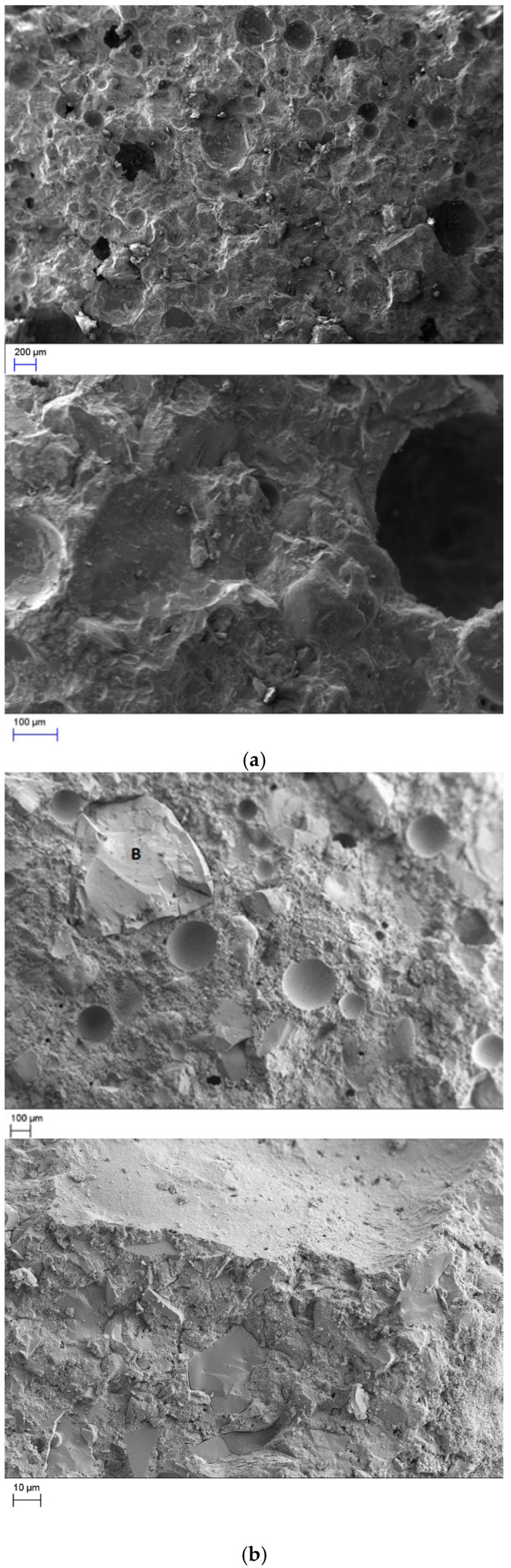

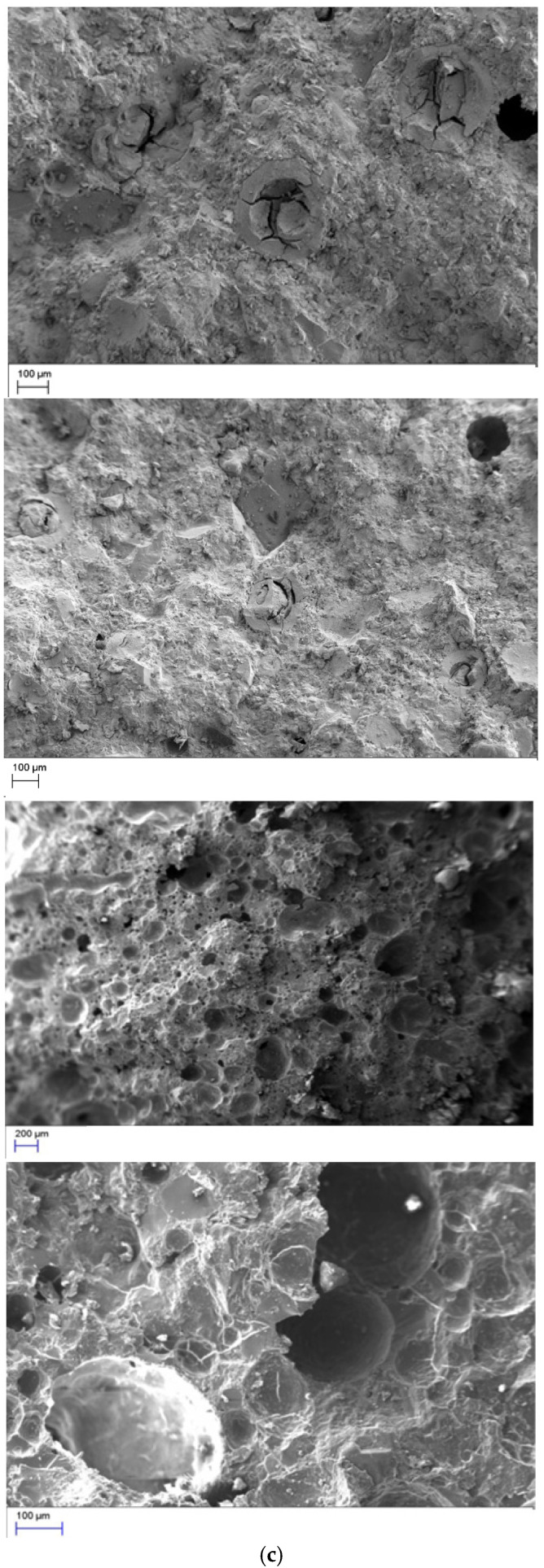
Microstructure of (**a**) the reference concrete mix (REF), (**b**) aerogel bead concrete mix (AC−10-(**b**), and (**c**) aerogel powder concrete mix (AC−10-P).

Some scanning electron microscope (SEM) analyses have been conducted in order to examine the microstructure of the different mixes. The Zeiss EVO LS 15 scanning electron microscope, which is manufactured by Carl Zeiss Ltd., was used for the analyses. The results are shown in Figure 8, where the microstructures of the 10% aerogel powder and 10% aerogel bead mixes are presented.

The reference mix (Figure 8a) shows a moderately porous material with a rough surface. In the mix with aerogel beads, the image features a denser matrix with well-defined, uniformly spherical pores distributed evenly throughout the material. This structure is indicative of a material designed for lightweight applications with controlled porosity. The smoother surface and fewer irregularities enhance the material’s thermal insulation properties.

In the aerogel powder mix, many microcracks and spherical-shaped cracked areas appear, which may have been created by drying shrinkage, which can explain the results presented in Figure 6. There are visible cracks and voids within some of the pores, suggesting potential structural weakness.

Comparing both aerogel mixes with the reference mix, it can be seen that the reference mix is more homogenous with less porous and fewer micro cracks. This is the reason why aerogel mixes have less compressive strength than the reference one.

## 4. Conclusions

This research is focused on the development of high-strength and thermally insulated concrete. Two different types of aerogel (powder and beads) as a partial substitution of sand have been used, and the results showed that a 30% replacement of sand (by volume) with aerogel beads could lead to a mix with a compressive strength of 70 MPa and thermal conductivity of 1.1 W/mK. Comparing this mix with the respective aerogel powder mix of this study and other 30% aerogel bead mixes from the literature, it can be seen that this mix has the highest compressive strength and the lowest thermal conductivity. The distribution of thermal conductivity was also examined, and it was found that aerogel bead mixes were not as homogeneous as the respective aerogel powder ones. This is a topic on which future research can be focused.

## Figures and Tables

**Table 1:** Aerogel concrete mix design [12].

Sample	Cement (kg/m^3^)	Silica Fume (kg/m^3^)	GGBS (kg/m^3^)	Sand (kg/m^3^)	Aerogel (kg/m^3^)	Water (kg/m^3^)	Superplasticizer (kg/m^3^)
REF	657	119	418	1051	0	185	59
AC−10-P AC−10-B	657	119	418	945.9	7.2	185	59
AC−20-P AC−20-B	657	119	418	840.8	14.4	185	59
AC−30-P AC−30-B	657	119	418	735.7	21.7	185	59

**Table 2:** Materials and their densities [10,12].

Material	Density (kg/m^3^)
Cement	3150
Silica fume	2200
GGBS	1900
Sand	1600
Aerogel	110

**Table 3:** Comparison between the current study and literature results.

	f_c_ in 0% Aerogel Mix	f_c_ in 30% Aerogel Mix	Drop in f_c_ (%)	Thermal Conductivity in 0% Aerogel Mix	Thermal Conductivity in 30% Aerogel Mix	Drop in Thermal Conductivity (%)
Powder	94	59	37.2	1.75	1.51	13.7
Beads	94	70	25.5	1.75	1.1	37.1
(T. Gao, 2014) [7]	62	30	52	2	1.05	47.5
(S. Ng, 2015) [8]	150	60	60	2.35	1.2	48.9

**Table 4:** Thermal conductivity (W/mK) measurements for each sample on the 14th day after casting.

	0.00%	10.00%		20.00%		30.00%	
Day 14 k (W/mK)		k (W/mK)		k (W/mK)		k (W/mK)	
		Powder	Beads	Powder	Beads	Powder	Beads
Point 1		1.791	0.78	1.727	1.44	1.648	1.25
Point 2	2.066	1.924	1.46	1.759	1.50	1.553	0.88
Point 3	1.955	1.905		1.635	1.24	1.583	1.13
Point 4	1.944	1.853	1.35	1.572	1.58	1.351	0.90
Average	1.99	1.87	1.20	1.67	1.44	1.53	1.04
CV	0.034	0.032	0.305	0.051	0.101	0.084	0.173

**Table 5:** Thermal conductivity (W/mK) measurements for each sample on the 28th day after casting.

	0.00%	10.00%		20.00%		30.00%	
Day 28 k (W/mK)		k (W/mK)		k (W/mK)		k (W/mK)	
		Powder	Beads	Powder	Beads	Powder	Beads
Point 1		1.655	1.82	1.569	1.60	1.579	
Point 2	1.675	1.705	1.56	1.597	1.24	1.534	
Point 3	1.761	1.47	0.95	1.577	1.52		
Point 4	1.823	1.707	1.54	1.648	0.68	1.416	
Average	1.75	1.63	1.47	1.60	1.26	1.51	1.1
CV	0.042	0.069	0.25064	0.022	0.33042	0.056	

## Data Availability

The original contributions presented in this study are included in the article. Further inquiries can be directed to the corresponding author.
